# ﻿Resin outpourings on conifers are inhabited by more members of Nectriaceae (Hypocreales, Sordariomycetes) than previously thought

**DOI:** 10.3897/mycokeys.113.140446

**Published:** 2025-02-12

**Authors:** Paweł Czachura, Paulina Janik

**Affiliations:** 1 W. Szafer Institute of Botany, Polish Academy of Sciences, Lubicz 46, PL-31-512 Kraków, Poland Polish Academy of Sciences Kraków Poland

**Keywords:** Morphology, multi-locus phylogeny, novel taxa, resinicolous fungi, taxonomy

## Abstract

Resin outpourings on conifers are a quite unique habitat occurring in nature. Conifer resins are composed mainly from monoterpenes, sesquiterpenes and resin acids which are recalcitrant to microbial decomposition. Moreover, resins exhibit antimicrobial properties. Despite that, they are colonised by different microorganisms including fungi. They are called resinicolous fungi. They constitute a poorly explored group of the fungal kingdom. In this study, during investigation of resinicolous fungi in Poland, seven strains were assigned to the family Nectriaceae. Phylogenetic analyses of combined ITS, LSU, *rpb2*, *tef1* and *tub2* sequence data were used for molecular identification. As a result, two new species (including a new genus) and two known species were identified. *Pulchrosporaresinae***gen. et sp. nov.** and *Cosmosporaelegans***sp. nov.** were described, characterised and proposed herein. Known species such as *Cosmosporaviridescens* and *Cosmosporellapruni* were isolated from resin substrate for the first time.

## ﻿Introduction

Resin outpourings arise as a consequence of physical damage or as response to infection of pathogens (Langenheim 2003; Cabrita 2018; Seyfullah et al. 2018). Resins play a major defensive role, they seal wounds and protect plants against external dangers such as insects, microbial infections, and others (Langenheim 2003; Cabrita 2018). Resins are also a quite unique substrate occurring in the natural environment. In conifers, resins are primarily composed of monoterpenes, sesquiterpenes and resin acids, as well as with addition of fatty acids, esters, sterols, alcohols, waxes and resenes (Langenheim 2003; Cabrita 2018). Moreover, due to their protective role against microorganisms, resins have toxic antifungal chemicals (Langenheim 2003; Cabrita 2018). All things considered, resin outpourings seem to be a harsh environment for microorganisms and recalcitrant to microbial decomposition. However, it is worth noting that there are some fungal taxa which can actively live on resins and are even specialised to such a substrate. They are called resinicolous fungi. Fungi reported from resin belong to many different lineages within the kingdom Fungi, best known are from orders: Helotiales, Leotiales, Mycocaliciales, Orbiliales and Xylonales (Tuovila et al. 2013; Beimforde et al. 2017; Baral et al. 2020; Beimforde et al. 2020; Hashimoto et al. 2021; Mitchell 2021; Mitchell et al. 2021). Interestingly, in spite of that, resinicolous fungi are highly scattered within the phylum Ascomycota, to date there are approximately 50 known species. Some of them belong to the family Nectriaceae (Hypocreales, Sordariomycetes) (Mitchell 2021). Nectriaceae was introduced by Tulasne and Tulasne (1865). Based on recent knowledge, the family consists of 77 genera (Perera et al. 2023) and includes fungi reported from terrestrial and aquatic habitats with diverse ecological lifestyles, mainly plant‐associated species such as saprobes on plant material, endophytes, plant pathogens, as well as fungicolous, lichenicolous, entomogenous species and animal pathogens (Lombard et al. 2015; Perera et al. 2023). There are also vague reports about resinicolous taxa (Corda 1837; Booth 1959; Mitchell 2021).

During broader research on resinicolous fungi in Poland, seven fungal strains were assigned to the family Nectriaceae based on preliminary molecular studies. The aim of this study is a detailed identification of analysed species based on morphology and multilocus phylogeny.

## ﻿Materials and methods

### ﻿Strains isolation

**Figure 1.**
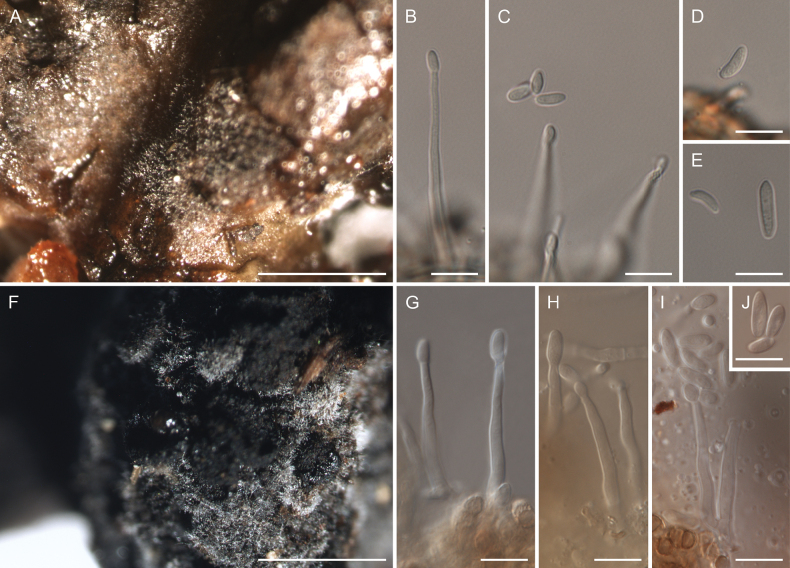
Fungal mycelium on resins **A–E** the specimen on a resin sample from which the strain CBS 152413 was isolated **F–J** the specimen on a resin sample from which the strain CBS 152414 was isolated **A** mycelium growing on resin **B, C** monophialidic conidiogenous cells with arising microconidia **D, E** microconidia **F** mycelium growing on resin **G, H** monophialidic conidiogenous cells with arising microconidia **I** monophialidic conidiogenous cells and microconidia **J** microconidia. Scale bars: 1 mm (**A, F**); 10 µm (**B–E, G–J**).

Resin samples were collected in three different locations in Poland – the Modrzyna Reserve, the Świętokrzyski National Park and the Tatra National Park. Samples were placed in sterile containers and secured. Strains analysed in this study were isolated in two different ways. Two strains (CBS 152413 and CBS 152414) were isolated from resin samples which were densely covered by an unknown fungus (Fig. 1). Mycelium visible to the naked-eye was isolated using a dissecting needle under a dissecting microscope. The five remaining strains were isolated in a different way. In a laboratory, resin samples were treated for removing external contaminants. The washing process was conducted using an orbital shaker operating at 200 rpm. Samples were washed in a sterile 0.1% solution of Tween 20 (Sigma - Aldrich, Munich, Germany) in physiological saline for 15 min. Then, resin samples were washed in sterile saline solution for 3 min. This process was repeated three times. Subsequently, the outer layer of resin was scratched using razor blades. Resin particles were spread on Petri dishes (Ø 90 mm) with four different media: dichloran – 18% glycerol agar (DG18), dichloran rose bengal chloramphenicol agar (DRBC), rose bengal chloramphenicol agar (RBC), potato dextrose agar (PDA). Mycological media were prepared accordingly with Crous et al. (2019) and Corry et al. (1995). Cultures with particles of resins had been incubated in the dark at 15 °C or 25 °C until fungal colonies appeared. Representatives of morphotypes were transferred to malt extract agar (MEA). One-month-old cultures were selected for a molecular analysis. Among numerous fungal taxa isolated by this method only members of the family Nectriaceae were selected for this study.

### ﻿Morphological analyses

Macroscopic features of cultures were studied on malt extract agar (MEA), oatmeal agar (OA), potato dextrose agar (PDA) and synthetic nutrient-poor agar (SNA) after 14 days at 15 °C and 25 °C in the dark. Mycological media for morphological characteristics were prepared accordingly with Crous et al. (2019). Colour notations were supported by the colour charts of Rayner (1970). The observations of fungal mycelia on resin samples were examined under a Nikon SMZ1500 dissecting microscope (Tokyo, Japan) equipped with a Nikon DS-Fi1 digital camera head.

Micromorphological features were examined from colonies after 14 days at 25 °C in darkness using slides or slide cultures, which were prepared accordingly with Crous et al. (2019). *Cosmosporaelegans* was examined on PDA and verified on MEA, OA and SNA. *Pulchrosporaresinae* was examined on SNA and verified on MEA, OA and PDA. Micromorphological features of fungal structures were examined by light microscopy using Nikon Eclipse E-600 compound light microscope equipped with a Nikon DS-Fi1 digital camera head. The measurements and photographs of fungal structures were conducted using imaging software NIS D-Elements v. 4.30 (Nikon).

### ﻿DNA extraction, amplification and sequencing

DNA was extracted from colonies grown on MEA medium after approximately 1 month. Extraction was performed using the CTAB method similarly to Gerrits van den Ende and de Hoog (1999) with minor modifications. About 1 cm^2^ of mycelium was scrapped and transferred to 1.5 ml tubes containing glass bulbs. The mycelium was disrupted by shaking on 1.5 Hz for 90 s. After first shaking, 500 μl CTAB isolation buffer was added (prepared accordingly with Doyle and Doyle (1987): 100 mM Tris-HCI, pH 8.0, 1.4 M NaCl, 20 mM EDTA, 2% CTAB, 0.2% 2-mercaptoethanol). Shaking on 1.5 Hz for 90 s was repeated twice. Between second and third shaking the tubes were incubated for 60 s on ice. Subsequently, samples were incubated in a water bath 65 °C for 10 min. After a water bath, 500 μl mixture of phenol, chloroform and isoamyl alcohol (at a ratio of 25:24:1) (Carl Roth GmbH + Co. KG, Germany) was added and vigorously vortexed. Tubes were centrifuged at 14000 rpm for 5 min. The supernatant of each sample was transferred to a new tube and 800 μl of 70% cold ethanol (stored at -20 °C) was added and mixed gently. Samples were stored at -20° for 30 min. After incubation, tubes were centrifuged at 14000 rpm for 5 min. Arisen pellets were rinsed with 500 μl of 70% cold ethanol (stored at -20 °C) and dried at 50 °C for 30 min. Finally, samples were suspended in 75 μl cell culture grade water (Sigma Aldrich, USA) and stored at -20 °C.

Five partial loci were amplified: the internal transcribed spacer 1 and 2 regions and intervening 5.8S rRNA gene (ITS), the 28S rRNA gene (LSU), the RNA polymerase II second largest subunit (*rpb2*) gene, the translation elongation factor 1-α (*tef1*) gene and the β-tubulin (*tub2*) gene. Amplification mixture for each sample contains: 17.5 μl cell culture grade water (Sigma Aldrich, USA), 2.5 μl of 10× PCR Buffer (Sigma Aldrich, USA), 2.5 μl of 25 mM MgCl_2_ (Sigma Aldrich, USA), 0.5 μl of 10 mM dNTPs (EURx, Poland), 0.5 μl of each 10 μM primers (Sigma Aldrich, USA), 0.25 μl of 5 U/μl Taq DNA Polymerase (Sigma Aldrich, USA) and 1 μl of isolated DNA. Amplifications were conducted using primers ITS1 and LR5 for a fragment containing ITS and LSU (White et al. 1990; Vilgalys and Hester 1990), fRPB2-5F and fRPB2-7cR for *rpb2* (Liu et al. 1999), EF1-728F and EF1-986R for *tef1* (Carbone and Kohn 1999), and T1 and T22 for *tub2* (O’Donnell and Cigelnik 1997). Amplification conditions for the fragment containing ITS and LSU were set as follows: an initial denaturation at 94 °C for 3 min, followed by 35 cycles of amplification (denaturation at 94 °C for 45 s; annealing at 50 °C for 45 s; elongation at 72 °C for 2 min), and a final elongation step at 72 °C for 10 min. Amplification conditions for parts of protein-coding genes were conducted as described by Piątek et al. (2024), Bensch et al. (2012), Nosratabadi et al. (2018) for *rpb2*, *tef1* and *tub2*, respectively. Amplification products were visualised on agarose gel electrophoresis. Finally, amplicons were enzymatically purified using Exo-BAP Mix (EURx, Poland) according to manufacturer protocol and prepared for bidirectional sequencing by Macrogen Europe B.V. (Amsterdam, The Netherlands). The ITS region was sequenced using primers ITS1 and ITS4 (White et al. 1990), whereas the LSU region was sequenced using primers LSU1Fd and LR5 (Vilgalys and Hester 1990; Crous et al. 2009). The remaining three loci – *rpb2*, *tef1* and *tub2* were sequenced with the same primer pairs that were used for their amplification.

### ﻿Phylogenetic analyses

Chromatograms were manually checked, then assembled and trimmed using Geneious Prime 2020.0.4. All newly obtained sequences were compared with sequences of other species existing in GenBank nucleotide database using the megablast search tool (Zhang et al. 2000) to reveal phylogenetic affinity of investigated fungal strains. Accession numbers for all sequences determined for this study and sequences retrieved from GenBank are listed in Table 1. The single-locus datasets were individually assembled and automatically aligned with the E-INS-i strategy in MAFFT v. 7.490 (Katoh et al. 2002; Katoh and Standley 2013). To detect phylogenetic concordance, single-gene trees were visually compared. With congruence, individual alignments of the ITS, LSU, *tub2*, *rpb2*, *tef1* were concatenated to build a multilocus matrix with SeaView (Gouy et al. 2010). Maximum likelihood (ML) and Bayesian inference (BI) analyses were used for estimating phylogenetic relationships. For both analyses, the best substitution models for each partition were determined with ModelTest-NG v. 0.2.0 (Flouri et al. 2014; Darriba et al. 2020) using the Akaike information criterion (AIC, Akaike 1974). Partitions were specified for ITS, LSU, *tef1*, as well as for the first, second and third codon positions of the *tub2* and *rpb2* exons, and for each of three non-coding regions of *tub2*. Substitution models selected for each partition for ML analysis were as follows: GTR+I+G4 for ITS and LSU; F81, F81+I, GTR+G4 for the first, second and third codon positions of *tub2*, respectively, as well as GTR+I+G4, HKY+I+G4, HKY+I+G4 for the first, second and third non-coding regions of *tub2*, respectively. Substitution models such as GTR+I+G4, HKY+I+G4, GTR+I+G4 were used for the first, second and third codon positions of *rpb2*, respectively, and the substitution model HKY+I+G4 was used for non-coding region of *tef1*. The BI analysis was performed with the following substitution models: GTR+I+G4 for ITS and LSU; F81, JC+I, GTR+G4 for the first, second and third codon positions of *tub2*, respectively, and SYM+I+G4, K80+I+G4, HKY+I+G4 for the first, second and third non-coding region of *tub2*, respectively. Substitution models such as GTR+I+G4, HKY+I+G4, GTR+I+G4 were used for the first, second and third codon positions of *rpb2*, respectively, and the substitution model HKY+I+G4 was used for non-coding region of *tef1*. ML analysis was conducted with RAxML-NG v. 1.2.2 (Kozlov et al. 2019) using non-parametric bootstrapping with 1 000 replicates. BI was performed using Metropolis-coupled Markov chain Monte Carlo (MCMCMC) method as implemented in MrBayes v. 3.2.3 (Ronquist et al. 2012) with the partitioning scheme and best-fit substitution model for each partition as described above. The phylogenetic analysis was run twice, each with four Metropolis chains for 10 million generations (sampling every 1000) under the best-fit substitution models with parameters unlinked across partitions. The first 25% of the sampled trees were discarded as a burn-in. Additionally, to reveal the phylogenetic position of the type of “*Cosmosporarishbethii*” (CBS 496.67) among the analysed group of species, a single-locus alignment of *rpb2* sequences with the sequence of “*Cosmosporarishbethii*” was also subjected to phylogenetic analyses (ML and BI). Both analyses followed the same settings used for multigene analyses. The best substitution model was GTR+I+G4 and SYM+I+G4 for ML and BI, respectively. The resulting trees were visualised with FigTree v. 1.4.2 (http://tree.bio.ed.ac.uk/software/figtree/) and graphically edited in CorelDRAW X7 (Ottawa, Canada).

**Table 1.** GenBank accession numbers and details of strains used in the phylogenetic analyses.

Species	Strain	Country	Isolation source	GenBank accession numbers				
				ITS	LSU	*rpb2*	*tef1*	*tub2*
*Cosmosporaarxii*	CBS 748.69 T	Germany	*Hypoxylon* sp.	KM231819	KM231694	HQ897725	KM231950	KM232089
*Cosmosporabutyri*	CBS 301.38 T = MUCL 9950	Denmark	Butter	MW827605	MW827644	HQ897729	–	–
*Cosmosporacoccinea*	CBS 341.70	Germany	*Inonotusnodulosus*	HQ897827	KM231692	HQ897777	KM231947	KM232086
*Cosmosporacylindricospora*	KUNCC 22–12662 T = CGMCC 3.24270	China	Submerged decaying wood in a stream	OP876700	OP872570	OQ077584	–	OQ025193
*Cosmosporacymosa*	CBS 762.69 IT	Germany	*Inonotusradiatus*	HQ897828	KM231693	HQ897778	KM231948	KM232087
***Cosmosporaelegans***	**CBS 152410 T**	**Poland**	**Resin of *Piceaabies***	**PQ484119**	**PQ484126**	**PQ474165**	**PQ474171**	**PQ474173**
„*Cosmosporaflavoviridis*”	IMI 338173	United Kingdom	Branch partially submerged in stream	KC291747	KC291785	–	–	KC291902
*Cosmosporafomiticola*	CBS 137813 T = G.J.S. 83-194	New Zealand	*Fomesfomentarius*	KJ676158	KJ676195	–	KJ676355	KJ676274
*Cosmosporainonoticola*	HMAS 248723 T	China	*Inonotus* sp.	KU563625	–	–	–	KU563621
*Cosmosporakhandalensis*	CBS 356.65 IT = IMI 112790	India	Decaying stem of *Bambusa* sp.	KJ676166	KJ676203	MW833997	KJ676368	KJ676287
*Cosmosporalavitskiae*	CBS 530.68 T = IMI 133984	Ukraine	Plant debris from rhizosphere soil	KJ676167	KJ676204	MW833998	KJ676369	KJ676288
„*Cosmosporaobscura*”	MAFF 241484	Japan	Twig	KC291719	KC291788	–	KC291858	KC291903
„*Cosmosporastegonsporii*”	CBS 122305 IT = A.R. 4385	Ukraine	*Stegonsporiumpyriforme*	KC291718	KC291755	–	KC291828	KC291901
„*Cosmosporastilbosporae*”	CBS 125509	Austria	Teleomorph of *Prostheciumellipsosporum*	MH863670	MH875134	–	–	–
„*Cosmosporastilbosporae*”	CBS 125512	Austria	Teleomorph of *Prostheciumellipsosporum*	MH863673	MH875137	–	–	–
*Cosmosporaviridescens*	IMI 73377a PT	Wales	*Ruzeniaspermoides*	KJ676171	KJ676208	–	KJ676373	KJ676292
*Cosmosporaviridescens*	A.R. 2783	Denmark	Bone	KJ676142	KJ676179	–	KJ676336	KJ676253
***Cosmosporaviridescens***	**CBS 152411**	**Poland**	**Resin of *Abiesalba***	**PQ484120**	**PQ484127**	**PQ474166**	**PQ474172**	**PQ474174**
„*Cosmosporaviridescens*”	CBS 102430	Czech Republic	Standing trunk of *Piceaabies*	KJ676147	KJ676184	–	KJ676342	KJ676260
„*Cosmosporaviridescens*”	CBS 102433	Czech Republic	*Tilia* sp., dead tree	KJ676148	KJ676185	MW833999	KJ676343	KJ676261
„*Cosmosporarishbethii*”	CBS 496.67 T	United Kingdom	*Pinussylvestris*, stump covered by resin	–	–	HQ897714	–	–
*Cosmosporellacavisperma*	CBS 172.31 ET = NRRL 13996	Norway	*Pinussylvestris*	MW827606	MW827645	MW834000	–	–
*Cosmosporellaolivacea*	KUMCC 17-0321 T	China	Dead wood	MH087212	MH087214	–	–	MH087216
*Cosmosporellaolivacea*	KUMCC 18-0016	China	Dead wood	MH087213	MH087215	–	–	MH087217
*Cosmosporellapruni*	MFLUCC 17-2579 T	Italy	Dead branch of *Prunusavium*	ON361570	ON352628	ON364492	ON364467	ON364480
***Cosmosporellapruni***	**CBS 152412**	**Poland**	**Resin of *Piceaabies***	**PQ484121**	**PQ484128**	–	**PQ474147**	**PQ474175**
*Dialonectriaepisphaeria*	CBS 125494	Canada	Ascomycete stromata	MH863609	MH875085	HQ897756	KM231953	KM232092
*Dialonectriafavaceae*	BRFM 2935 T	France	Dead stromata of Diatrypellacf.favacea	MW198213	MW198211	MW558054	–	–
*Dialonectriamagnusiana*	BRFM 1591	France	Diatrypellacf.favacea	MW198212	MW198210	–	–	–
*Dialonectriavolutella*	CBS 125493	USA	Ascomycete on *Fagusamericana*	KM231821	KM231696	HQ897782	KM231952	KM232091
„*Fusariummelanochlorum*”	CBS 202.65	Austria	*Fagussylvatica*	MH858541	MH870179	HQ728162	–	–
*Fusicollaaquaeductuum*	CBS 837.85 ET = BBA 64559 = NRRL 20865	Germany	Plug in water tap	KM231823	KM231699	HQ897744	KM231955	KM232094
*Fusicollamatuoi*	CBS 581.78 = ATCC 18694 = NRRL 20427	Japan	*Albizziajulibrissin*	KM231822	KM231698	HQ897720	KM231954	KM232093
*Fusicollaquarantenae*	CBS 141541 T = URM 8367	Brazil	*Melocactuszehntneri*	MW553789	MW553788	MW556626	MW556625	MW556624
*Fusicollaviolacea*	CBS 634.76 T = BBA 62461 = NRRL 20896	Iran	*Quadraspidiotusperniciosus*	KM231824	KM231700	HQ897696	KM231956	KM232095
*Macroconiabulbipes*	CBS 146679 T = CPC 37138	South Africa	*Erica* sp. associated with *Dimerosporiopsisengleriana*	MW827617	MW827657	MW834018	–	MW834310
*Macroconiapapilionacearum*	CBS 125495 = DAOM 238119	USA	Ascomycete on Fabaceae	HQ897826	KM231704	HQ897776	KM231958	KM232096
*Macroconiaphlogioides*	CBS 146501 T = CPC 35389	South Africa	Leaf of *Encephalartos* sp.	MW827620	MW827660	MW834020	–	MW834313
*Macroconiasphaeriae*	CBS 717.74	France	Stromata of pyrenomycete	KM231827	KM231707	KM232390	JF735695	KM232099
*Microceracoccophila*	CBS 310.34 = NRRL 13962	Italy	Scale insect	HQ897794	KM231703	HQ897705	JF740692	–
*Microceralarvarum*	CBS 738.79 ET = BBA 62239 = MUCL 19033 = NRRL 20473	Iran	*Quadraspidiotusperniciosus*	KM231825	KM231701	KM232387	KM231957	–
*Microceralichenicola*	CBS 149169 T = CPC 41114	Netherlands	*Parmeliasulcata*	ON811502	ON811561	–	–	ON803591
*Microcerarubra*	CBS 638.76 T = BBA 62460 = NRRL 20475	Iran	*Quadraspidiotusperniciosus*	HQ897820	KM231702	HQ897767	JF740696	EU860019
Nectriacinnabarina	CBS 125165 = A.R. 4477 ET	France	Dead twigs of *Aesculus* sp.	HM484548	HM484562	KM232402	HM484527	HM484606
„*Nectriaflavoviridis*”	CBS 124353 = BBA 65542	USA	Decorticated wood	HQ897791	MW827664	HQ897702	–	–
*Nectriamariae*	CBS 125294 = A.R. 4274 T	France	*Buxussempervirens*	JF832629	JF832684	KM232404	JF832542	JF832899
*Pseudocosmosporabeijingensis*	CGMCC 3.24131 T	China	Rotten bark, associated with other fungi	OP223438	OP223434	–	–	OP272862
*Pseudocosmosporaeutypae*	CBS 133961 T = C.H. 11-01	France	*Eutypa* sp.	KC291735	KC291766	–	KC291837	KC291925
*Pseudocosmosporaeutypellae*	CBS 133966 T = A.R. 4562	USA	*Eutypella* sp.	KC291721	KC291757	–	KC291830	KC291912
*Pseudocosmosporarogersonii*	CBS 133981 T = G.J.S. 90-56	USA	*Eutypella* sp.	KC291729	KC291780	–	KC291852	KC291915
*Pseudocosmosporavilior*	CBS 133971 ET = A.R. 4810	Argentina	*Eutypella* sp.	KC291737	KC291763	–	KC291834	KC291928
***Pulchrosporaresinae***	**CBS 152413 T**	**Poland**	**Resin of *Abiesalba***	**PQ484122**	**PQ484129**	**PQ474167**	**PQ474148**	**PQ474176**
***Pulchrosporaresinae***	**CBS 152414**	**Poland**	**Resin of *Abiesalba***	**PQ484123**	**PQ484130**	**PQ474168**	**PQ474149**	**PQ474177**
***Pulchrosporaresinae***	**CBS 152415**	**Poland**	**Resin of *Piceaabies***	**PQ484124**	**PQ484131**	**PQ474169**	**PQ474150**	**PQ474178**
***Pulchrosporaresinae***	**CBS 152416**	**Poland**	**Resin of *Piceaabies***	**PQ484125**	**PQ484132**	**PQ474170**	**PQ474151**	**PQ474179**

Sequences and details of strains obtained in this study are shown in bold. Abbreviations: A.R.: Collection of Amy Y. Rossman, USDA-ARS, MD, USA; ATCC: American Type Culture Collection, Manassas, VA, USA; BBA: Biologische Bundesanstalt für Land- und Forstwirtschaft, Institut für Mikrobiologie, Berlin, Germany; BRFM: Bank of Fungal Resources of Marseille, Marseille, France; C.H.: Collection of Cesar S. Herrera, University of Maryland, MD, USA; CBS: Westerdijk Fungal Biodiverity Institute, Utrecht, The Netherlands; CGMCC: China General Microbiological Culture Collection Center, Beijing, China; CPC: Culture collection of Pedro Crous, housed at the Westerdijk Fungal Biodiversity Institute, Utrecht, Netherlands; DAOM: Canadian National Mycological Herbarium and Culture Collection, AAFC, Ottawa, Ontario, Canada; G.J.S.: Collection of G.J. Samuels, USDA-ARS, MD, USA; HMAS: Herbarium Mycologicum Academiae Sinicae, Chinese Academy of Sciences, Beijing, China; IMI: CABI Bioscience, Egham, UK; KUMCC: Kunming Institute of Botany Culture Collection, Kunming, China; KUNCC: Kunming Institute of Botany Culture Collection, Kunming, China; MAFF: Ministry of Agriculture, Forestry and Fisheries, Tsukuba, Ibaraki, Japan; MFLUCC: Mae Fah Luang University Culture Collection, Chiang Rai, Thailand; MUCL: Mycotheque delUniversite Catholique de Louvain, Louvain-la-Neuve, Belgium; NRRL: Agricultural Research Service Culture Collection, National Center for Agricultural Utilization Research, USDA, Peoria, IL, USA; URM: Micoteca do Departmento de Micologia, Universidade Federal de Pernambuco, Recife, Brazil; ET: ex-epitype; IT: ex-isotype; PT: ex-paratype; T: ex-holotype; – indicates unavailable data or sequence. A name inside quotation marks refers to the misidentified taxonomic affiliation.

## ﻿Results

### ﻿Phylogenetic analyses

**Figure 2.**
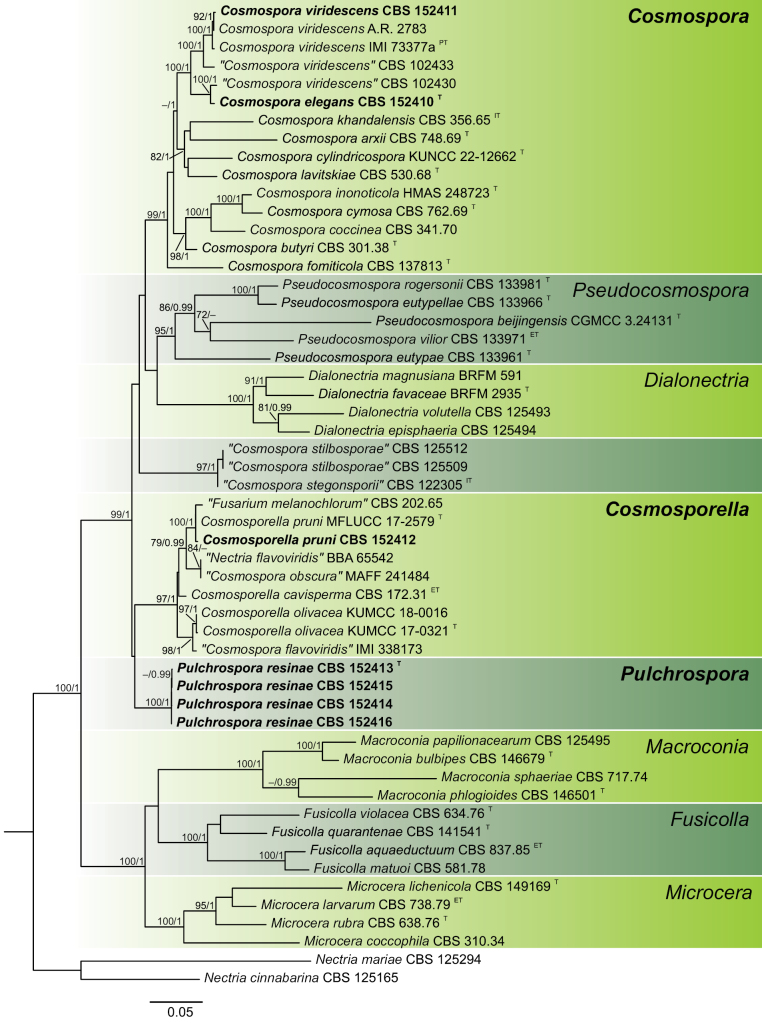
Maximum likelihood consensus tree inferred from the combined ITS, LSU, *tub2*, *rpb2* and *tef1* multiple sequence alignment of selected members of Nectriaceae. Maximum likelihood bootstrap (MLB) support values ≥ 70% and Bayesian posterior probabilities (BPP) ≥ 0.9 are given above branches (MLB/BPP). The positions of studied strains are indicated in bold. Ex-type, ex-epitype, ex-isotype, ex-paratype strains are indicated with T, ET, IT, PT respectively. A name inside quotation marks refers to the misidentified taxonomic affiliation. The scale bar represents the average number of substitutions per site.

The multi-gene alignment length was 3 508 bp including gaps, for five gene regions. The phylogenetic analyses included 52 ingroup taxa, with *Nectriamariae* (CBS 125294) and *Nectriacinnabarina* (CBS 125165) as outgroup taxa. The phylogenetic placement of analysed strains was performed by multilocus analysis of ITS, LSU, *tub2*, *rpb2* and *tef1* and presented in Fig. 2. The tree topology obtained from the ML analysis was confirmed by the Bayesian phylogenetic tree. Maximum likelihood bootstrap (MLB) support values above 70% and Bayesian posterior probabilities (BPP) above 0.9 were given above branches.

In general, both phylogenies are characterised by mostly strong statistical support for external branches and poor supporting values for internal branches, and therefore the relationships between analysed genera are mostly not resolved. Sequences of the strain CBS 152411 clustered with the paratype specimen of *Cosmosporaviridescens* (IMI 73377a) with fully support (MLB = 100%, BPP = 1), whereas sequences of the strain CBS 152412 clustered with fully support (MLB = 100%, BPP = 1) with the type specimen of *Cosmosporellapruni*. Sequences of remaining strains did not cluster with any of the known type species, and therefore they are considered as novel taxa.

### ﻿Taxonomy

#### ﻿ Cosmospora elegans

Taxon classificationFungiHypocrealesNectriaceae

Czachura & Janik sp. nov.

1C81298F-FFDF-5945-AEF2-1CDC09641FEC

856972

Figs 3 , 4

##### Etymology.

The name refers to the visually attractive appearance of cultures of this species.

##### Typus.

Poland, Małopolskie Province, Tatra County, the Tatra National Park, Dolina Białego, isolated from the resin of *Piceaabies*, 16. Jul. 2021, leg. P. Czachura (holotype: KRAM F-60000; culture ex-type: CBS 152410).

##### Description.

Mycelium consisting of branched, septate, hyaline to subhyaline or yellowish orange in mass, smooth hyphae, 1.7–4.2 µm diam., with occasional anastomoses. Chlamydospores rarely formed, subglobose to broadly ellipsoidal, hyaline to subhyaline, smooth, aseptate, 8.1–13.1 × 6.1–9.5 µm, intercalary or terminal, single. Conidiophores arising laterally or terminally from somatic hyphae, 20.2–74.7 μm long, unbranched or branched, hyaline, smooth- and thin-walled, bearing terminal and lateral conidiogenous cells, or reduced to single conidiogenous cells borne laterally or terminally on aerial hyphae. Conidiogenous cells monophialidic, subulate or subcylindrical, hyaline, smooth- and thin-walled, 20.2–55.4 μm long, at the base 2.0–3.3 μm wide and at the apex 1.2–1.9 μm wide, without noticeable periclinal thickening, collarettes absent or sometimes a minute apical collarette can be present. Microconidia ellipsoidal, cylindrical, slightly allantoid or obovoid to clavate, hyaline, smooth- and thin-walled, aseptate, 3.5–14.7 × 2.3–3.8 μm.

**Figure 3.**
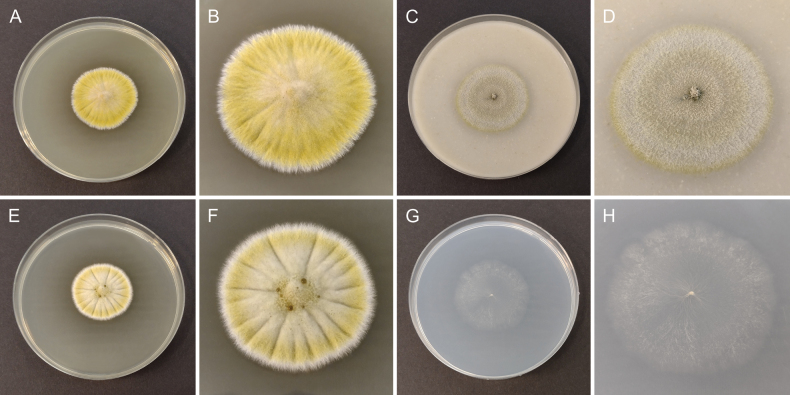
Morphology of cultures of *Cosmosporaelegans* (CBS 152410) after 2 weeks of growth at 25 °C **A, B** colony on MEA**C, D** colony on OA**E, F** colony on PDA**G, H** colony on SNA.

##### Culture characteristics.

Colony on MEA umbonate with fimbriate margin, slightly radially folded from the colony centre toward margin, yellow with white margin, reaching 21 mm diam. after 2 weeks at 15 °C and 35 mm diam. after 2 weeks at 25 °C, reverse luteous. Colony on OA flat with slightly fimbriate margin, whitish to creamy with yellowish margin, reaching 21 mm diam. after 2 weeks at 15 °C and 39 mm diam. after 2 weeks at 25 °C, reverse fuscous black to pale brown at margin. Colony on PDA umbonate with fimbriate margin, radially folded from the colony centre toward margin, creamy at centre becoming yellow toward the outer part of colony with white margin, reaching 20 mm diam. after 2 weeks at 15 °C and 34 mm diam. after 2 weeks at 25 °C, reverse bright luteous with whitish margin. Colony on SNA flat with slightly undulate and slightly fimbriate margin, whitish, reaching 22 mm diam. after 2 weeks at 15 °C and 40 mm diam. after 2 weeks at 25 °C, reverse whitish.

**Figure 4.**
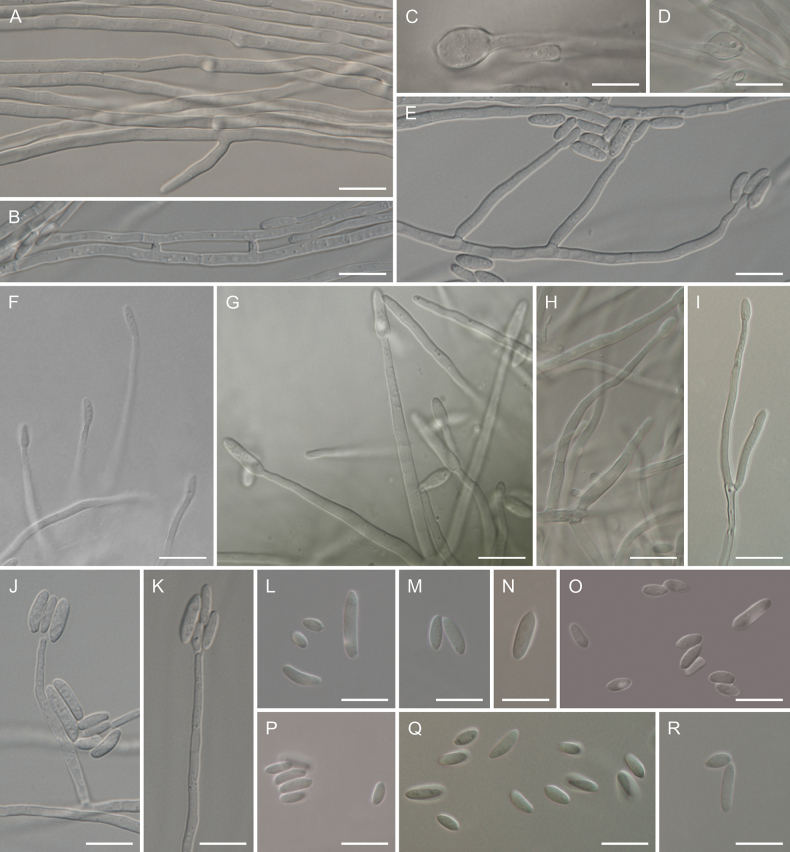
Morphology of *Cosmosporaelegans* (CBS 152410) **A** hyphae **B** anastomosing hyphae **C, D** chlamydospores **E–K** conidiophores and monophialidic conidiogenous cells with arising microconidia **L–R** microconidia. Scale bars: 10 µm (**A–R**).

##### Notes.

*Cosmosporaelegans* clusters as a fully-supported lineage (MLB = 100%, BPP = 1) to the closest phylogenetic relative – *Cosmosporaviridescens* (Fig. 2), from which it can be differentiated mainly by the size and the shape of conidia. *C.elegans* has more elongated conidia in shape (mostly ellipsoidal and cylindrical) and larger in size (3.5–14.7 μm) in contrast to *C.viridescens* whose conidia are globose or reniform and reaching 4–8 μm long (Booth 1959). Additionally, *C.elegans* has smaller conidiophores which reach 74.7 μm in length in the type specimen whereas conidiophores of *C.viridescens* reach 500 μm (Booth 1959). Moreover, *C.elegans* rarely forms single chlamydospores in contrast to *C.viridescens* in which chlamydospores are absent (Booth 1959).

#### ﻿ Pulchrospora

Taxon classificationFungiHypocrealesNectriaceae

Czachura & Janik gen. nov.

987440E8-8FCD-5FDA-9CEE-0B06D85E442C

856973

##### Etymology.

The name refers to the beautiful shape of macroconidia of this genus.

##### Description.

Mycelium consisting of branched, septate, hyaline, smooth hyphae. Aerial conidiophores unbranched or branched, hyaline, bearing terminal and lateral conidiogenous cells, or reduced to single conidiogenous cells borne laterally or terminally on aerial hyphae. Conidiogenous cells monophialidic, subcylindrical, cylindrical or slightly subulate, hyaline, often with a conspicuous flared collarettes, without noticeable periclinal thickening. Microconidia ellipsoidal, cylindrical, slightly reniform or obovoid, hyaline, smooth, aseptate or septate. Sporodochial conidiophores irregularly and verticillately branched, bearing lateral and terminal solitary monophialides. Sporodochial conidiogenous cells monophialidic, slightly subulate to subcylindrical, hyaline, collarettes absent or inconspicuous, without noticeable periclinal thickening. Sporodochial macroconidia falcate, slightly curved to curved with parallel walls, regularly wide along almost the entire length, tapering in apical cell toward the apex (rarely tapering towards both ends), apical cell slightly curved to curved, basal cell obtuse, non foot-shaped, aseptate or septate, hyaline, smooth. Chlamydospores unknown.

##### Type species.

*Pulchrosporaresinae* Czachura & Janik.

#### ﻿ Pulchrospora resinae

Taxon classificationFungiHypocrealesNectriaceae

Czachura & Janik sp. nov.

71F33282-AE2F-57DF-B77E-6C5B8021920A

856974

Figs 5 , 6

##### Etymology.

The name refers to the habitat of this species.

##### Typus.

Poland, Podkarpackie Province, Krosno County, the Modrzyna Reserve, isolated from the resin of *Abiesalba*, 21. Jun. 2021, leg. P. Czachura (holotype: KRAM F-60003; culture ex-type: CBS 152413).

##### Description.

Mycelium consisting of branched, septate, hyaline, smooth hyphae, 1.5–5.5 µm diam., with frequent anastomoses. Aerial conidiophores arising laterally or terminally from somatic hyphae, 16.8–68.4 μm long, unbranched or branched, hyaline, smooth- and thin-walled, bearing terminal and lateral conidiogenous cells, or more commonly reduced to single conidiogenous cells borne laterally or terminally on aerial hyphae. Conidiogenous cells monophialidic, subcylindrical, cylindrical or slightly subulate, hyaline, smooth- and thin-walled, 16.8–48.5 μm long, at the base 2.2–3.6 μm wide and at the apex 1.3–2.0 μm wide, often with conspicuous flared collarettes, without noticeable periclinal thickening. Microconidia forming small false heads, ellipsoidal, cylindrical, slightly reniform or obovoid, hyaline, smooth- and thin-walled, aseptate or rarely 1-septate, 3.8–21.6 × 2.4–4.5 μm. Sporodochial conidiophores densely packed, irregularly and verticillately branched, bearing lateral and terminal solitary monophialides. Sporodochial conidiogenous cells monophialidic, slightly subulate to subcylindrical, hyaline, smooth-walled, 17.2–32.2 μm long, at the base 2.4–3.3 μm wide and at the apex 1.5–2.2 μm wide, collarettes absent or inconspicuous, without noticeable periclinal thickening. Sporodochial macroconidia falcate, slightly curved to curved with parallel walls, regularly wide along almost the entire length, tapering in apical cell toward the apex (rarely tapering towards both ends), apical cell slightly curved to curved, basal cell obtuse, non-foot-shaped, 1–5-septate (rarely aseptate or 6–7-septate), hyaline, smooth, 14.3–44.8 × 3.1–4.8 μm.

**Figure 5.**
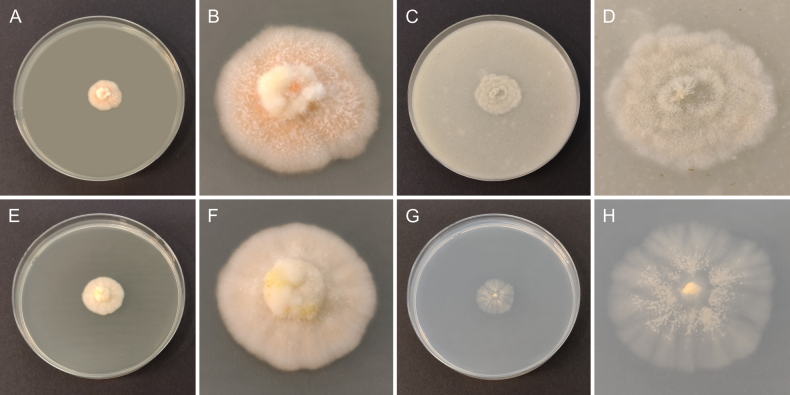
Morphology of cultures of *Pulchrosporaresinae* (CBS 152413) after 2 weeks of growth at 25 °C **A, B** colony on MEA**C, D** colony on OA**E, F** colony on PDA**G, H** colony on SNA.

##### Culture characteristics.

Colony on MEA umbonate with slightly undulate margin, rosy buff with white aerial mycelium, reaching 12 mm diam. after 2 weeks at 15 °C and 19 mm diam. after 2 weeks at 25 °C, reverse pale buff. Colony on OA flat with crenate margin, whitish, reaching 18 mm diam. after 2 weeks at 15 °C and 25 mm diam. after 2 weeks at 25 °C, reverse whitish to slightly pale yellow. Colony on PDA umbonate with slightly undulate margin, pale buff to pale rosy buff, reaching 11 mm diam. after 2 weeks at 15 °C and 22 mm diam. after 2 weeks at 25 °C, reverse pale buff. Colony on SNA flat with crenate margin, whitish, reaching 11 mm diam. after 2 weeks at 15 °C and 21 mm diam. after 2 weeks at 25 °C, reverse whitish.

**Figure 6.**
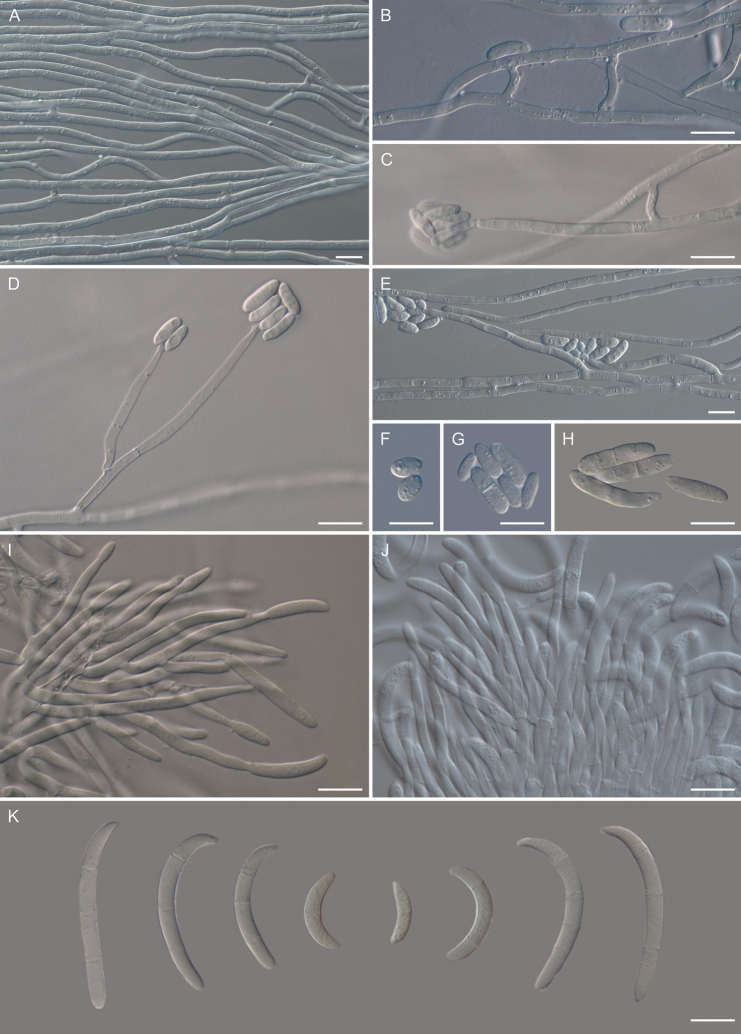
Morphology of *Pulchrosporaresinae* (CBS 152413). **A** hyphae **B** anastomosing hyphae **C–E** conidiophores and monophialidic conidiogenous cells with arising microconidia **F–H** microconidia **I, J** sporodochial conidiophores and conidiogenous cells with arising macroconidia **K** macroconidia. Scale bars: 10 µm (**A–K**).

##### Additional specimens examined.

Poland, Podkarpackie Province, Krosno County, the Modrzyna Reserve, isolated from the resin of *Abiesalba*, 22. Jun. 2021, leg. P. Czachura (KRAM F-60004; culture CBS 152414); Poland, Podkarpackie Province, Krosno County, the Modrzyna Reserve, isolated from the resin of *Piceaabies*, 23. Oct. 2020, leg. P. Czachura (KRAM F-60005; culture CBS 152415); Poland, Świętokrzyskie Province, Kielce County, the Świętokrzyski National Park, the strict protection area Psarski Dół, isolated from the resin of *Piceaabies*, 16. Oct. 2020, leg. P. Czachura (KRAM F-60006; culture CBS 152416).

##### Notes.

*Pulchrosporaresinae* gen. et sp. nov. is phylogenetically closest to the genera *Cosmospora*, *Cosmosporella*, *Dialonectria* and *Pseudocosmospora*. All these genera formed the highly supported clade (MLB = 99%, BPP = 1) but with unresolved relationship between genera in this group (Fig. 2). *Pulchrosporaresinae* produces micro- and macroconidia similarly to *Dialonectria* and *Cosmosporella*. However, *P.resinae* produces microconidia on aerial conidiophores and macroconidia are produced on sporodochial conidiophores, like in the genus *Dialonectria*, but differently from the genus *Cosmosporella* whose members produce micro- and macroconidia on aerial conidiophores (Huang et al. 2018; Crous et al. 2021; Perera et al. 2023). Moreover, the general morphology of macroconidia between these genera differs significantly. Macroconidia of *P.resinae* are characterised by being regularly wide along almost the entire length, tapering toward the apex (mostly) and obtuse, non-foot-shaped base, in contrast to members of *Dialonectria* and *Cosmosporella*, whose macroconidia are characterised by slightly tapering towards both ends and having foot-shaped basal cells (Crous et al. 2021). Additionally, macroconidia of *Cosmosporella* are commonly irregularly wide and longer (50–90 μm) than macroconidia of *P.resinae* which measured 14.3–44.8 μm (Gerlach and Nirenberg 1982; Crous et al. 2021).

## ﻿Discussion

In this study seven strains represented by four species were identified as belonging to three genera of Nectriaceae: *Cosmospora*, *Cosmosporella* and the newly established *Pulchrospora*. The genus *Cosmospora* was erected by Rabenhorst (1862). In the past, the genus was treated in a broad sense (Rossman et al. 1999) without molecular data and to date accumulated 84 “*Cosmospora*” names (www.mycobank.org; www.indexfungorum.org). However, during further, more accurate molecular studies, most of them were transferred to other genera. In recent years, based on molecular analyses the genus concept was restricted to species closely related with the type species *Cosmosporacoccinea* (later named *Cosmospora* sensu stricto). Based on studies conducted by authors in recent years (Gräfenhan et al. 2011; Herrera et al. 2015; Zeng and Zhuang 2016; Luo et al. 2019; Lechat and Fournier 2021; Bao et al. 2023), 20 species are considered as reliable species of *Cosmospora* in *Cosmospora* sensu stricto genus concept. Species belonging to *Cosmospora* sensu stricto are characterised by acremonium-like asexual morphs (producing microconidia) and live mostly on other fungi (Gräfenhan et al. 2011). Other species such as *C.khandalensis*, *C.lavitskiae*, *C.ustulinae* and *C.viridescens* were also reported from fungi but also as saprobes on plants, wood, decaying wood, rhizosphere soil and even bone (Teng 1934; Booth 1959; Sukapure and Thirumalachar 1966; Zhdanova 1966; Gräfenhan et al. 2011; Herrera et al. 2015). Moreover, two recently discovered species of *Cosmospora*: *C.cylindricospora* and *C.aquatica* are only known on decaying wood submerged in water (Luo et al. 2019; Bao et al. 2023). These examples show that members of this genus are not exclusively specialised for living on other fungi. In this study, *C.viridescens* and a new species *C.elegans* were isolated from conifer resins. It is a new niche for members of *Cosmospora* sensu stricto. Interestingly, both species belong to a small group of *Cosmospora* members which are saprobes on different substrates, mostly plant material. To date, *C.viridescens* was known from occurring on another fungus *Ruzeniaspermoides* and from bone (Gräfenhan et al. 2011; Herrera et al. 2015), and in this study, we found a specimen of *C.viridescens* from a new habitat for this species. It is worth mentioning that there are two additional strains in the CBS culture collection (CBS 102430 and CBS 102433) previously considered as *C.viridescens*. However, in a study conducted by Herrera et al. (2015) authors used these strains for a phylogenetic analysis and mentioned that these strains may represent a different species closely related to *C.viridescens*. Our study confirmed that suggestion. In the present study, the strain from resin (CBS 152410) and the strain CBS 102430 clustered together (Fig. 2). The phylogenetic and morphological analyses revealed that strain CBS 152410 from resin is morphologically and phylogenetically distinct from the type of *C.viridescens*. Based on phylogenetic analyses conducted in this study (Fig. 2), the strain CBS 102430 presumably represents another strain of *C.elegans*, which was isolated from a standing trunk of *Piceaabies*.

Apart from members of *Cosmospora*, we found a member of the genus *Cosmosporella* in this study. The genus *Cosmosporella* was introduced by Huang et al. (2018) and currently it comprises three formally described species – *Cosmosporellaolivacea*, *Cosmosporellacavisperma* and *Cosmosporellapruni* (Huang et al. 2018; Crous et al. 2021; Perera et al. 2023). However, phylogenetic analyses indicate that the genus may consist of more species which need a formal description, which was shown on Fig. 2 and in the study conducted by Perera et al. (2023). Nevertheless, all strains clustered within the lineage of the genus *Cosmosporella*, which were isolated from plant materials (Table 1). Interestingly, the type specimen of *C.cavisperma* (described as *Fusariumcavispermum*) was found on resin by Corda (1837). However, molecular data from the type specimen was not available and, therefore, the species was epitypified by Crous et al. (2021). The isolation of *C.pruni* in our study shows that living on resin is a wider adaptation within the genus *Cosmosporella* to this ecological niche. *C.pruni* was previously found only on a fallen dead branch of *Prunusavium* (Perera et al. 2023). This study extends the knowledge about this species that it may be also associated with conifers.

Analysed sequences of *Pulchrosporaresinae* formed a distinct lineage within the family Nectriaceae (Fig. 2). Moreover, the lineage of *P.resinae* did not cluster with sufficient support to any of the known genera. Additionally, the species differs from closest relatives by morphological features and ecology. All things considered, *P.resinae* is here recognised as the novel genus within the family Nectriaceae. Based on phylogenetic inference *P.resinae* clustered together with genera *Cosmospora*, *Cosmosporella*, *Dialonectria* and *Pseudocosmospora* in a highly supported clade (MLB = 99%, BPP = 1). However, the phylogenetic relationship between these genera remains unresolved (Fig. 2), which corresponds with other studies conducted in this group (Herrera et al. 2013; Huang et al. 2018; Crous et al. 2021; Perera et al. 2023). However, it is worth mentioning that each genus in this branch formed a well-supported genus clade (MLB = 99%, BPP = 1 for *Cosmospora*; MLB = 97%, BPP = 1 for *Cosmosporella*; MLB = 100%, BPP = 1 for *Dialonectria*; MLB = 95%, BPP = 1 for *Pseudocosmospora*). To sum up, an unresolved position of *P.resinae* corresponds to a general scheme observed in this group that phylogenetic relationships between genera are unresolved but generic clades are well-supported. Additionally, morphological differences between *P.resinae* and the remaining genera in this clade have proved that *Pulchrospora* is a distinct genus. In this clade, *Cosmospora* and *Pseudocosmospora* represent acremonium-like asexual morph in contrast to *Cosmosporella*, *Dialonectria* and *Pulchrospora* which represent fusarium-like asexual morphs (Herrera et al. 2013; Crous et al. 2021). Moreover, there are morphological differences between fusarioid genera (see notes under *P.resinae*). In addition to these morphological differences, the genera *Cosmospora*, *Dialonectria* and *Pseudocosmospora* differ from *P.resinae* by a great phylogenetic distance and ecology – their members are mainly fungicolous, even highly host speciﬁc (Herrera et al. 2013, 2015, 2016; Sun et al. 2019; Zeng and Zhuang 2022). Despite the lack of stability in the phylogenetic position of *P.resinae*, multilocus sequence analysis showed that the genus is closely related to *Cosmosporella*. It corresponds to the biology of both genera whose members are saprobes on plant material, mainly on dead wood, trees and resin of conifers, in contrast to the rest of genera in this clade which members are mainly fungicolous. However, both genera exhibit different morphology (see notes under *P.resinae*), which is another argument for recognising *P.resinae* as a new genus.

To date, two members of Nectriaceae were well documented from resins – *Cosmosporellacavisperma* and “*Cosmosporarishbethii*” (Corda 1837; Cappelletti 1927; Booth 1959; Rossman et al. 1999; Crous et al. 2021; Mitchell 2021). Comparing *Pulchrosporaresinae* with *Cosmosporellacavisperma* (described as *Fusariumcavispermum*), it is worth noting that *C.cavisperma* produced macroconidia on resin (Corda 1837). It differs this species from the newly erected *P.resinae* which produced exclusively phialides and microconidia in two separate resin samples (Fig. 1). The second species reported from resin was “*Cosmosporarishbethii*”. The species was isolated from a *Pinussylvestris* stump, having perithecia immersed in secreted resin (Booth 1959). In a study conducted by Gräfenhan et al. (2011), the type specimen of “*Cosmosporarishbethii*” clustered near the group analysed in this study but with weak support. To exclude a phylogenetic relationship between *P.resinae* and “*Cosmosporarishbethii*”, we included the ex-type strain of “*Cosmosporarishbethii*” (CBS 496.67) in our preliminary phylogenetic analysis. However, the strain clustered outside of our analysed clade, therefore it was excluded from the final phylogenetic analysis. For visualisation that both species are phylogenetically different taxa, a phylogenetic tree inferred from *rpb2* sequences is given (Suppl. material 1). Based on this analysis, “*Cosmosporarishbethii*” constitutes a new phylogenetic lineage in Nectriaceae.

In this study, two new species of Nectriaceae were described based on its asexual morph. It is worth mentioning that the morphology of asexual morphs is often crucial for distinguishing taxa in this group (Lombard et al. 2015). Finally, based on multilocus phylogenetic analysis, four species of Nectriaceae are reported from resin for the first time. This study extends our knowledge about the ecology of Nectriaceae and the diversity of resinicolous fungi as well. Plant resins undoubtedly seem to be a unique habitat occurring in nature and therefore scientific interest in microorganisms living on them should be increased. Detecting and describing new species of resinicolous fungi is important to understand these intriguing organisms.

## Supplementary Material

XML Treatment for Cosmospora elegans

XML Treatment for Pulchrospora

XML Treatment for Pulchrospora resinae

## ﻿Additional information

### Author ORCIDs

Paweł Czachura https://orcid.org/0000-0002-3562-8776

Paulina Janik https://orcid.org/0000-0002-4106-636X

### Data availability

All of the data that support the findings of this study are available in GenBank nucleotide database (https://www.ncbi.nlm.nih.gov/genbank/), the culture collection and the fungal herbarium, as shown in Table 1 and the main text.

## Supplementary materials

Supplementary material 1 Maximum likelihood consensus tree inferred from *rpb2* sequence alignment of selected members of Nectriaceae Data type pdf Explanation note Maximum likelihood bootstrap (MLB) support values ≥ 70% and Bayesian posterior probabilities (BPP) ≥ 0.9 are given above branches (MLB/BPP). The position of studied strains are indicated in bold. Ex-type, ex-epitype, ex-isotype, ex-paratype strains are indicated with T, ET, IT, PT respectively. A name inside quotation marks refers to the misidentified taxonomic affiliation. The scale bar represents the average number of substitutions per site. https://binary.pensoft.net/file/1249096This dataset is made available under the Open Database License (http://opendatacommons.org/licenses/odbl/1.0/). The Open Database License (ODbL) is a license agreement intended to allow users to freely share, modify, and use this Dataset while maintaining this same freedom for others, provided that the original source and author(s) are credited.Paweł Czachura, Paulina Janik
