# Supplementary figures and images for: ﻿Resin outpourings on conifers are inhabited by more members of Nectriaceae (Hypocreales, Sordariomycetes) than previously thought
Source: MycoKeys. 2025 Feb 12;113:337–58. doi: 10.3897/mycokeys.113.140446 (PMC11840427; doi:10.3897/mycokeys.113.140446)

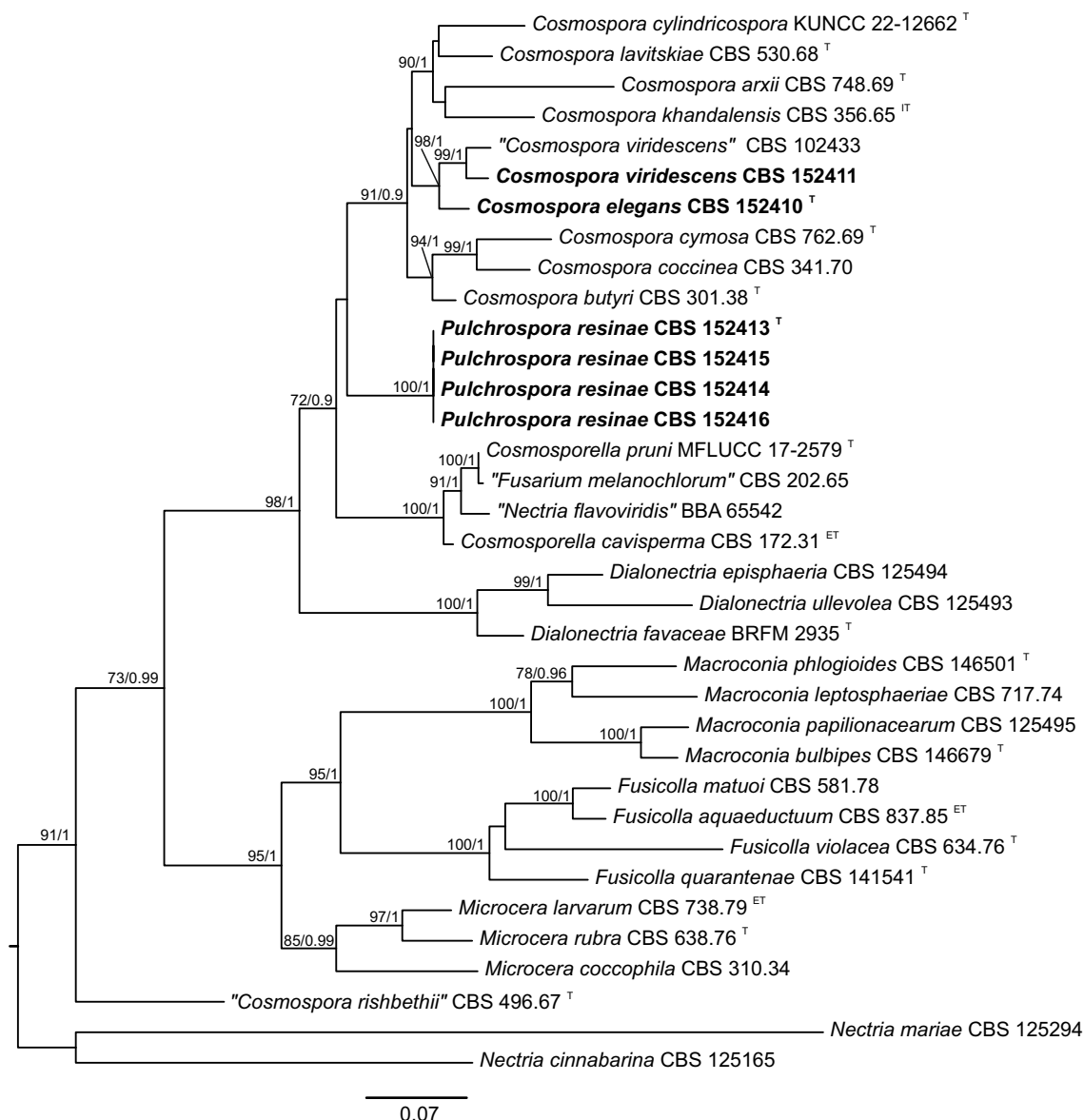

Supplement: Supplementary material 1 — Maximum likelihood consensus tree inferred from rpb2 sequence alignment of selected members of Nectriaceae [file mycokeys-113-337-s001.pdf]
